# Editorial: Perceptions of People: Cues to Underlying Physiology and Psychology

**DOI:** 10.3389/fpsyg.2020.00643

**Published:** 2020-04-08

**Authors:** Danielle Sulikowski, Kok Wei Tan, Alex L. Jones, Lisa L. M. Welling, Ian D. Stephen

**Affiliations:** ^1^Perception and Performance Research Group, School of Psychology, Charles Sturt University, Bathurst, NSW, Australia; ^2^School of Psychology and Clinical Language Sciences, University of Reading Malaysia, Gelang Patah, Malaysia; ^3^Department of Psychology, College of Human and Health Sciences, Swansea University, Swansea, United Kingdom; ^4^Psychology Department, Oakland University, Rochester, MI, United States; ^5^Department of Psychology, Macquarie University, Sydney, NSW, Australia; ^6^Perception in Action Research Centre, Macquarie University, Sydney, NSW, Australia; ^7^Body Image and Ingestive Behaviour Group, Macquarie University, Sydney, NSW, Australia

**Keywords:** mate quality signals, health, fertility, formidability, person perception

Our perceptual sensitivity to cues of socially and sexually relevant physiological and psychological traits in others is remarkable. For such sensitivity to evolve, the directly perceptible qualities of others (which include intrinsic physical traits, such as height, weight, body odor, facial morphology, and body shape; as well as behaviorally modified appearance cues, such as those produced by clothing and makeup; and vocal parameters) must afford at least somewhat accurate judgements of others‘ health, fertility, formidability, personality, or other fitness-relevant capacities. The current issue examines whether a variety of perceptible qualities present potentially valid cues of underlying physiology and psychology, and/or the extent to which such cues support adaptive judgements of others.

The human face is a highly complex signaling system, and not surprisingly, it features heavily in this Research Topic. Sexual dimorphism (masculinity in male faces and femininity in female faces) is one on the most studied aspects of facial morphology (for review, see, e.g., Little et al., 2011) and its importance in mate relevant choices is again highlighted in the current issue. Facial morphological correlates of bodily muscle mass in male faces predict female perceptions of facial masculinity; and such correlates are perceived as more attractive for short-term relationships, and less attractive for long-term relationships by said women (Lei et al.). Chen et al.. present a meta-analysis of preferences for (primarily) facial sexual dimorphism, confirming well-reported associations between sexual dimorphism and perceived attractiveness. Preferences for highly sexually dimorphic partners are thought to be condition-dependent, reflecting a trade-off made by higher quality individuals, which compromises kind, caring personalities (especially in less masculine men) in favor of more sexually dimorphic, genetically robust individuals. Consistent with this theory, Chen et al. also report a reliable (though small) positive association between own attractiveness and preferences for sexual dimorphism (especially in women rating men as long-term partners, and men rating women as short-term partners). Štěrbová et al. reported that women's long-term partner preferences are generally highly stable from one relationship to the next, with one exception: facial masculinity. The facial masculinity of participants' long-term partners varied more than expected by random coupling—the only trait of 21 measured to do so. In addition, long-term partners with whom the participants had children also had more masculine faces than non-fathers.

Beyond sexual dimorphism, facial adiposity, and skin color are also important cues to physical and physiological health. Facial adiposity is a highly reliable indicator of body mass index and numerous health outcomes (most notably those associated with being overweight or obese; de Jager et al.). Across cultures and ethnicities, skin yellowness (which indicates the presence antioxidant carotenoids), and to a lesser extent skin redness (an indicator of blood flow associated with physical fitness and levels of sex hormones) are subjectively perceived by observers as indicators of physical health (Tan and Stephen). Cues to physiological health, especially anaerobic capacity, may also be present in human faces. Judgements of the fighting ability of mixed martial arts fighters, based on 360° headshots, were jointly predicted by the fighters' weight and anaerobic capacity (Trebick et al.). Although this study took advantage of the additional information available in a 360° headshot compared to a single front-on portrait, Trebick et al. also demonstrated that attractiveness and formidability judgements of front-on, profile, and 360° portraits differ very little in terms of their means, and are strongly inter-correlated. This observation bolsters many conclusions drawn from across the field of evolutionary psychology of face perception, many of which are based on judgements of single front-on portraits.

The judgments we make of human faces may also influence how we perceive non-human primate faces. Primate faces that are least like human faces are perceived as the most beautiful (Rádlová et al.), an effect attributed to the uncanny valley (i.e., the hypothesized relationship between resemblance to humans and emotional response; e.g., Mori et al., 2012). The more like human faces primate faces become, however, the more that human-face predictors of attractiveness judgements (in terms of the exact arrangements of the internal features), also predicted the beauty judgements of the primate faces.

New data-driven methods for analyzing facial morphology have also been presented in this Research Topic. Mogilski and Welling present a novel application of data-driven conjoint analysis showing that eyebrow thickness, jaw prominence, and facial height exhibit superior signaling capacity for judgements of sexual dimorphism and attractiveness compared to other regions of the face. Kleisner et al. proposed a new method for measuring cultural typicality and cross-cultural distinctiveness of faces. Based on calculating an individual face's exact position on the vector connecting the facial morphological averages of the two cultures being compared, Kleisner et al. were able to predict significant variance in faces' perceived distinctiveness. They also demonstrated that in-group/out-group judgements were easier (in a two-alternative forced-choice design) when the out-group face was furthest away from the in-group mean. Both of these novel methodologies open the door for more nuanced analyses of the signaling capacity of human facial morphology.

Non-morphological, behavioral cues, including vocalizations and body odor, also provide information about their bearer. Single men, compared to partnered men, exhibit stronger body odor, and based on their body odor alone are judged by women to be more masculine (Mahmut and Stevenson). Listeners also adjust their perceptions of another based on the pitch of vocalizations. Mixed martial arts fighters are judged to be more formidable if their roars (spontaneous vocalizations made when victorious) are higher pitched, and also if their speaking voice is lower pitched (Šebesta et al.). Similarly contrasting effects of pitch are observed when comparing singing and speaking voices. The perceived attractiveness of singing and speaking strongly correlate across individuals, and both predict physical size in men (Valentova et al.). Also in men, low-pitched speech, but higher-pitched singing, predict higher sociosexuality. In women, shorter apparent vocal tract length (indicated by shorter spacing between the first four formant frequencies) in speech, but longer apparent vocal tract length during singing, predicted higher sociosexuality (Valentova et al.). It therefore appears that both pitch and range of vocalizations provide important cues as to the quality of potential mates and competitors.

Extended phenotypic traits, manifesting beyond the bearer's physical body, may also constitute socially meaningful cues. Two such adornments include clothing and make-up. Gouda-Vossos et al. investigated how wearing business vs. casual attire influenced perceptions of men's and women's socio-economic status. Business attire increases the perceived economic status of men more than it does for women, while the perceived economic status of women is increased if depicted in business attire alongside a group of men (whereas the economic status of men is not increased by being depicted among a group of women). The consequences of such judgements for interpersonal interactions are not known. Women use cosmetics to alter their physical appearance and this can affect their perceived attractiveness. Batres et al. observed that women using the contraceptive pill spend less time putting on make-up for an outing than do naturally cycling women, and that such women are indeed perceived as wearing more make-up. This finding adds to the breadth of behaviors in which circulating sex hormones are implicated (see Welling and Shackelford, 2019), although the complexities of female cosmetics use, what it signals, and how it is perceived remain to be thoroughly investigated.

The methods used by evolutionary psychologists to understand how the multitude of physical and behavioral cues we possess are signaled and received were also a focus of critique within this special issue (see Bovet; Kleisner et al.; Trebick et al.). Bovet reviewed the literature concerning women's waist-to-hip ratios and criticized the weak theoretical foundations of the area. Highlighting the large number of potential signaling functions of the waist-to-hip ratio and the small number of studies designed to specifically differentiate between the competing theories, Bovet cautions against rushing into empirical work without first establishing a clear theoretical basis to guide empirical robustness and consistency. Doing so can lead to imprecise, untestable predictions and seemingly contradictory results, leading to the premature rejection of potentially legitimate theories based on findings of objectively low evidentiary value.

Cues and signals of physiology, behavior, and personality exist in faces, bodies, voices and our extended phenotypes. For many such potential cues, we have demonstrated objective links between the putative cue and the underlying quality it may indicate; observed receiver sensitivity to such cues; or put forward coherent adaptive and mechanistic theories accounting for how such cues may come to signal these qualities in the first place (in either a proximate or ultimate sense). For this area of person perception to continue to move forward, concerted efforts are needed to address all three of the above outcomes for each individual cue-quality-receiver system. By triangulating objective relationships between cues and qualities, the impact of such cues on receiver psychology, and sophisticated mechanistic, functional, and adaptive theories to explain the evolution and maintenance of these systems, we will amass a body of work with great potential impact for understanding modern human social, romantic, and sexual interactions.

## Author Contributions

DS was the primary author of the first draft. All authors contributed to revisions and corrections of the final manuscript.

### Conflict of Interest

The authors declare that the research was conducted in the absence of any commercial or financial relationships that could be construed as a potential conflict of interest.
